# Network-Based Analysis of Schizophrenia Genome-Wide Association Data to Detect the Joint Functional Association Signals

**DOI:** 10.1371/journal.pone.0133404

**Published:** 2015-07-20

**Authors:** Suhua Chang, Kechi Fang, Kunlin Zhang, Jing Wang

**Affiliations:** Key Laboratory of Mental Health, Institute of Psychology, Chinese Academy of Sciences, Beijing, China; University of Illinois at Chicago, UNITED STATES

## Abstract

Schizophrenia is a common psychiatric disorder with high heritability and complex genetic architecture. Genome-wide association studies (GWAS) have identified several significant loci associated with schizophrenia. However, the explained heritability is still low. Growing evidence has shown schizophrenia is attributable to multiple genes with moderate effects. In-depth mining and integration of GWAS data is urgently expected to uncover disease-related gene combination patterns. Network-based analysis is a promising strategy to better interpret GWAS to identify disease-related network modules. We performed a network-based analysis on three independent schizophrenia GWASs by using a refined analysis framework, which included a more accurate gene *P*-value calculation, dynamic network module searching algorithm and detailed functional analysis for the obtained modules genes. The result generated 79 modules including 238 genes, which form a highly connected subnetwork with more statistical significance than expected by chance. The result validated several reported disease genes, such as *MAD1L1*, *MCC*, *SDCCAG8*, *VAT1L*, *MAPK14*, *MYH9* and *FXYD6*, and also obtained several novel candidate genes and gene-gene interactions. Pathway enrichment analysis of the module genes suggested they were enriched in several neural and immune system related pathways/GO terms, such as neurotrophin signaling pathway, synaptosome, regulation of protein ubiquitination, and antigen processing and presentation. Further crosstalk analysis revealed these pathways/GO terms were cooperated with each other, and identified several important genes, which might play vital roles to connect these functions. Our network-based analysis of schizophrenia GWASs will facilitate the understanding of genetic mechanisms of schizophrenia.

## Introduction

Schizophrenia (SZ) is a severe psychiatric disorder affecting **~**5‰ of the population worldwide [1, 2]. Family and twin studies have shown schizophrenia has high heritability ranging 73%–90% [3]. Genome-wide association study (GWAS) is an effective strategy to screen disease related genetic factors at genome level [4, 5]. Plenty of GWASs have been conducted for schizophrenia and have identified many susceptibility loci [6–12], but they could only explain a small proportion of the genetic component of schizophrenia. The underlying genes remain largely unknown, especially the interplays between these susceptibility genes. It has been commonly accepted that the combination effects of multiple loci with moderate statistical significance, which might be lost in the GWAS approach due to the stringent significance level after multiple comparison correction, might make a risk contribution to schizophrenia.

To detect the common polygenic variations contributed to schizophrenia, analyses that focus on pathways or gene combination patterns could facilitate the reveal of disease related genes and the underlying genetic basis [13]. Pathway-based analysis (PBA) is one type of method employed to identify trait-associated pathways from GWAS [13] and has been used in many psychiatric diseases [14–16], autoimmune diseases [17, 18] *etc*. However, PBA depends on prior biological pathways and treats the whole pathway as a single unit, it cannot detect a small portion of the pathway or other new combination of genes which may be associated with disease. Recent years, network-based analysis, which was originally used for gene expression data [19], was proposed to analyze GWAS data [20] and has been applied into several diseases with different analysis framework [21–24]. This method aims to identify connected network modules that show significant disease association signals by integrating GWAS data with molecular network. Compared with pathway-based analysis, the molecular network used in network-based analysis has higher coverage of human genes and the resulted modules could be of any size [20].

The Psychiatric Genetic Consortium (PGC) has collected a wealth of valuable genotypic data for schizophrenia, while the traditional single marker analysis will miss a large proportion of weakly associated markers. Network-based analysis of these data would facilitate the discovery of novel susceptibility genes or modules for schizophrenia. Despite there have been three network-based analyses for schizophrenia [21, 24, 25], the analysis pipeline still needs to further refine to sufficiently explore valuable information from the large number of genotyping data. Firstly, considering the collections of GWAS data from PGC were genotyped using various platforms and the sample sizes varied largely, how to effectively in-depth mine these data requires continuous exploration to obtain new knowledge. Secondly, it has been shown that gene *P*-value calculation is a critical step in network-based analysis [25]. Several gene-based association test methods have been developed and they have better performance than minimal *P*-value of SNPs in one gene, such as VEGAS [26] and GATES [27]. Furthermore, besides detecting the combined pattern of multiple genes relevant to schizophrenia, deeply functional annotation and analysis of the identified module genes could provide new insights for the understanding of disease mechanism.

In this study, we performed a network-based analysis on three independent schizophrenia GWASs from PGC by using a more accurate gene *P*-value calculation [26], dynamic network module searching algorithm [20] and detailed functional analysis for the obtained module genes. The result generated 79 modules including 238 genes, which formed a highly connected subnetwork with more statistical significance than expected by chance. The result validated several reported disease genes, such as *MAD1L1*, *MCC*, *SDCCAG8*, *VAT1L*, *MAPK14*, *MYH9* and *FXYD6*, and also obtained several novel candidate genes and gene-gene interactions. Pathway enrichment analysis for the module genes showed they were significantly enriched in several neural and immune system related signaling pathways, biological processes, and protein activities. Furthermore, crosstalk analysis of these pathways assisted the understanding of the functional connections between them and revealed several important genes, such as *MAPK1*, *PSMB8*, *TRAF6* and *CAV1*, which might play vital roles during these functions. Our network-based analysis of schizophrenia GWASs would facilitate the understanding of genetic mechanism of schizophrenia.

## Materials and Methods

### GWAS datasets and preprocessing

The GWAS data used in this study was from PGC and approved by the National Institute of Mental Health (NIMH) (https://www.nimhgenetics.org/available_data/data_biosamples/gwas_data.php). Six collections of GWAS data genotyped on Affymetrix 6.0 platform and three collections of GWAS data genotyped on Affymetrix 500K platform were selected for this study. Then, we merged these datasets into three groups and named them as MGS, Affy6, and Affy500K separately. The detailed information about the GWAS datasets was summarized in Table 1. A consistent quality control process was conducted for the three GWAS datasets separately using PLINK [28]. Briefly, (i) SNPs were excluded if they had a missing rate >0.05 (before sample removal below); (ii) we removed samples with missing rate >0.05 or heterozygosity rate outside 3 standard deviation from the mean; (iii) we calculated identity by descent (IBD) after pruning SNPs using *r* ^*2*^ = 0.2 as threshold, and then excluded duplicated or related individuals with IBD>0.185; and (iv) we excluded SNPs with significant call rate difference in cases and controls (*P*<10^−5^), minor allele frequency (MAF) <0.05 or Hardy-Weinberg equilibrium *P*<10^−6^. Principal component estimation was done with EIGENSTRAT [29], which used the same collection of SNPs for IBD calculation. We estimated the first 20 principle components and evaluated their impact on the genome-wide test statistics using λ as [11]. Based on this, we used the top five principle components with the smallest λ together with the study indicator variables as associated covariates for logistic regression test to calculate *P*-values of SNPs. To check the *P*-value of identified module genes in a larger dataset, SCZ2 was applied [30]. SCZ2 SNP *P*-value list was downloaded from PGC website (http://www.med.unc.edu/pgc/downloads) and filtered to keep only SNPs whose imputation info score > 0.8. More than eight million SNPs were left for subsequent analysis.

**Table 1:** Schizophrenia GWAS datasets used for this analysis.

Collection	Country	Platform	Cases^b^	Controls^b^	SNPs^b^
**MGS**	United States, Australia	Affymetrix 6.0	**2681 (2679)**	**2653 (2653)**	**638,937 (576,742)**
**Affy6**			**1805 (1800)**	**2351 (2327)**	**541,356 (490,100)**
ISC-Cardiff	Bulgaria	Affymetrix 6.0	527	609	654,278
ISC-Dublin	Ireland	Affymetrix 6.0	272	860	642,723
ISC-Edinburgh	UK	Affymetrix 6.0	368	284	646,310
ISC-SW2	Sweden	Affymetrix 6.0	390	229	661,602
SGENE-TOP3	Norway	Affymetrix 6.0	248	369	620,195
**Affy500K**			**1078 (1058)**	**3591 (3509)**	**203,009 (182,499)**
Cardiff UK^a^	UK	Affymetrix 500K	476	2,938	362,569
CATIE	United States	Affymetrix 500K; Perlegen 164K	410	391	384,528
Zucker Hillside	United States	Affymetrix 500K	192	190	258,470

^a^ The controls of Cardiff UK were from WTCCC [69].

^b^ The number of cases, controls and SNPs after quality control were labeled in parentheses.

### Protein-protein interaction network dataset

The protein-protein interaction (PPI) network data was collected from InWeb [31], which contains high-confidence interactions as those seen in multiple independent experiments and reported more often in lower-throughput experiments. After mapping with approved gene symbol in HGNC [32] and removing self-interactions, the remaining PPI network was comprised of 12,387 proteins and 156,095 interactions.

### Gene-based association analysis

In the network analysis pipeline, the gene-based association analysis was conducted using VEGAS [26], which takes into account both the effects of all SNPs mapped to gene and linkage disequilibrium (LD) between SNPs by using simulations from the multivariate normal distribution to calculate the gene *P*-values. VEGAS allocates SNPs to one or more autosomal genes according to gene positions, in which gene version of hg18 was used and LD patterns of SNPs were estimated using HapMap data. Herein, the offline version of VEGAS was used. All SNPs mapped to gene within 20kb upstream and downstream of gene coordinates were used to calculate the gene *P*-value. In addition, several improvements were made based on the downloaded version. First, the SNP positions of three GWAS datasets and gene version were updated to GRCh37.p11 and downloaded from Ensembl BioMarts [33]; second, all genes with HGNC symbols were filtered to ensure at least one SNP was mapped within 20kb of gene coordinates; third, the SZ specific genotype data was used to estimate LD patterns for SNPs within each gene instead of the HapMap data. Adjust gene *P*-values was calculated using *p*.*adjust* function in R by inputting gene *P*-value list as input and setting adjust method as Benjamini & Hochberg (FDR). To calculate gene *P*-value from the largest scale of GWAS data SCZ2 for replication, we used an alternative gene-based association test tool, *i*.*e*. GATES [27], which does not need permutation or simulation to evaluate empirical significance and is much faster. 1000 Genome phase I [34] EUR genotype data (hg19) were used for LD calculation. Extended gene region length was 20kb at both 5’ and 3’. Threshold of *r* ^*2*^ for SNPs in high LD was 0.8. Other parameters were default.

### Network module search and evaluation

We used a dense module search (DMS) method, which is a R package developed by Jia *et al*. (called dmGWAS) [20], to identify functional modules enriched for SZ association signals. The DMS algorithm dynamically searches for a dense module that holds as many genes with small *P*-value as possible for each node in the context of a node-weighted PPI network. The module score is defined as Zm=∑zik, where k is the number of genes within a module, *z*_*i*_ is transferred from gene *P*-value according to *z*_*i*_ = ∅^−1^(1 − *P*_*i*_), in which ∅^−1^ denotes the inverse normal distribution function [19]. In each round of module searching, the DMS algorithm starts with each seed gene as the initial module and identifies neighborhood interactors, which are defined as nodes whose shortest path to the module is within a distance *d*. The genes generating the maximum increment of *Z*_*m*_ will be added to the module if *Z*_*m*+1_ > *Z*_*m*_(1 + *r*), where *Z*_*m*_ is the original module score, *Z*_*m*+1_ is the new module score, and *r* is a pre-defined expansion rate. Herein, *d* and *r* were set to 2 and 0.1 as [20]. This process iterates until none of the nodes can satisfy *Z*_*m*+1_ > *Z*_*m*_(1 + *r*). To evaluate the significance of the identified modules, we undertook two steps of examination. First, we calculated *P*-values based on module score (*Z*_*m*_) for each module by empirically estimating the null distribution [35]. Specifically, module scores *Z*_*m*_ were first median-centered by subtracting the median value of *Z*_*m*_ from each of them (*Z*_*median*_). Then, the mean δ and standard deviation σ for the empirical null distribution were estimated using *locfdr* in R packages. The module scores were standardized by Zs=Zmedian−δσ and converted to *P*-values by *P*(*Z*_*m*_) = 1 − ∅(*Z*_*S*_), where ∅ is the normal cumulative density function. Second, we made a cross evaluation among three GWAS datasets using ‘dualEval’ function in the dmGWAS package to minimize the bias from different GWAS datasets [20]. Details of cross evaluation are provided in dmGWAS document. Briefly, the modules from one GWAS dataset were used as discovery dataset and the modules’ significance in the other two GWAS datasets, which were as evaluation datasets, were evaluated in turn (*i*.*e*. *P*(*Z*_*m(eval)*_) was calculated). The criteria used to screen modules were the modules with *P*(*Z*_*m*_)<0.05 in the discovery dataset and *P*(*Z*_*m(eval)*_)<0.05 in any of the two evaluation datasets. Finally, the modules passed the tests from all three datasets were merged together and denoted as the resultant modules. The workflow of the network-based analysis for SZ GWAS data was shown in S1 Fig.

### Statistical and functional analysis of module genes

DAPPLE (Disease Association Protein-Protein Link Evaluator) [36] was used with input of a list of genes to evaluate how closely connected between the proteins encoded by module genes. Number of permutation was set to 10,000 times and common interactor binding degree cutoff was set to 2.

We used the DAVID Gene Set Enrichment Analysis tool [37, 38] to evaluate the enriched functions of identified module genes, including GO biological process, GO cellular component, GO molecular function [39] and KEGG pathways [40]. Only terms with an adjusted *P*-value (Benjamini & Hochberg) of less than 0.05 and with gene numbers less than 350 were retained as [41].

## Results

### Gene-based tests

We calculated gene *P*-values for the three SZ GWASs datasets using VEGAS with some refinements. The result was shown in Table 2. In order to perform network-based analysis, each node in the PPI network was assigned a gene *P*-value as the node weight if the name was matched. Accordingly, a MGS-weighted PPI consisting of 11,456 genes, an Affy6-weighted PPI consisting of 11,362 genes, and an Affy500K-weighted PPI consisting of 10,232 genes were generated, and these three weighted PPIs were denoted as background sets for the follow-up network analysis. There were 651 (5.7%), 681 (6.0%), and 589 (5.8%) genes with *P*-value < 0.05 in the background sets of MGS, Affy6 and Affy500K separately.

**Table 2:** Results for gene-based test and dynamic module search of three schizophrenia GWAS datasets.

GWAS datasets		MGS	Affy6	Affy500K
No. of genes	Total genes	27,188	26,899	23,420
	Genes in PPI	11,456	11,362	10,232
	Genes in PPI and *P*-value < 0.05	651 (5.7%)	681 (6.0%)	589 (5.8%)
	Genes in PPI and FDR < 0.6	56 (0.49%)	6 (0.05%)	46 (0.45%)
No. of modules	Initial dmGWAS modules	10,910	10,786	9,481
	① evaluation, *P*(*Z*_*m*_) < 0.05	786	123	612
	② evaluation in Affy6, *P*(*Z*_*m*(*eval*)_) < 0.05	17	-	41
	③ evaluation in Affy500K, *P*(*Z*_*m*(*eval*)_) < 0.05	11	19	-
	④ evaluation in MGS, *P*(*Z*_*m*(*eval*)_) < 0.05	-	34	5
	Final modules: ①∩(②∪③∪④)	27	6	46
Involved genes (238)		68	29	146
Genes with *P*-value < 0.05		34 (50%)	17 (58.6%)	83 (56.8%)
Genes with FDR < 0.6		9 (13.24%)	6 (20.69%)	15 (10.27%)

### Disease related network modules

**Fig 1:**
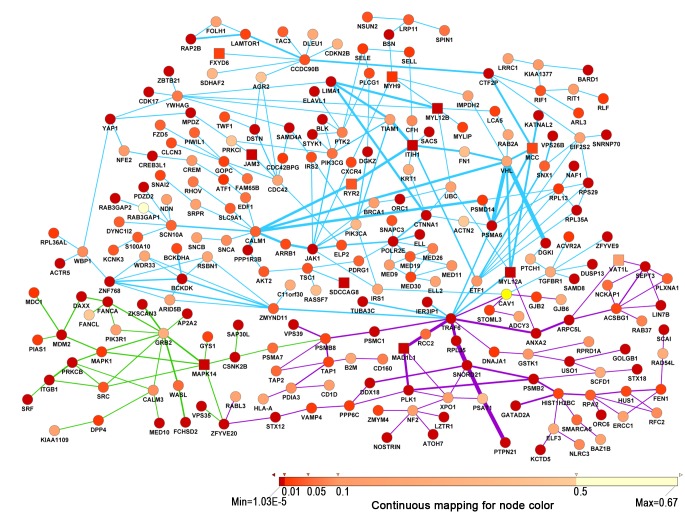
Protein-protein interaction network involving all merged module genes. Square nodes denote the reported genes associated with schizophrenia or bipolar disorder. The color of the node was proportioned with the *P*-value of gene. The width of the edge was proportioned with the No. of repeats of the edge in the modules. The purple edges, green edges and blue edges were interactions from MGS, Affy6 and Affy500K respectively.

We next sought to identify modules enriched for SZ association signals in the background PPIs using dmGWAS. By performing dense module search with each gene in the background PPI as a seed, 10,910 modules for MGS, 10,786 modules for Affy6, and 9,481 modules for Affy500K were initially obtained (Table 2). After examining the significance of module score and cross validation, 27 modules (including 68 genes) for MGS, 6 modules (including 29 genes) for Affy6 and 46 modules (including 146 genes) for Affy500K met the criteria. Furthermore, compared with the proportion of genes with *P*-value < 0.05 contained in the background PPI sets, the identified module genes for three datasets have a higher proportion of genes with *P*-value < 0.05 (as shown in Table 2). After merging all modules from three datasets, a total of 79 disease related modules were obtained and they formed a resultant subnetwork (Fig 1), which was composed of 238 module genes (S1 Table) and 317 interactions (S2 Table) between the genes in the same module. In this subnetwork, two genes were shared between MGS and Affy6 (*PSMB8* and *ZFYVE20*), and three genes were shared between MGS and Affy500K (*TRAF6*, *MYL12A* and *CAV1*). In addition, some interactions were overrepresented in different modules, such as *RPL35—SNORD21—PSAT1—PTPN21* from MGS, *FCHSD2—WASL—GRB2*—*MAPK14* from Affy6, *PSMA6—VHL—DGKI* and *TIAM1- ITIH1* from Affy500K.

### Annotation and functional analysis of module genes

To annotate the module genes which had been reported to be associated with schizophrenia, we searched two genetic databases: GWAS Catalog [42] and SZGene [43], and found that four genes (*MAD1L1*, *SDCCAG8*, *MCC* and *VAT1L*) had been reported their susceptibility with SZ by GWAS [11, 44–46], and three genes (*MAPK14* [47], *MYH9* [48] and *FXYD6* [49]) had been reported at least one positive association in SZGene. Since many evidence suggested that schizophrenia and bipolar disorder (BD) share some symptoms and genetic factors [50], we also searched these genes in the bipolar disorder genetic database BDgene [51]. The result showed that 6 genes (including *MAD1L1* [52], *JAM3* [53], *MYL12B* [53], *MYL12A [53]*, *ITIH1* [52] and *RYR2* [54]) had been reported to be significant associated with bipolar disorder in at least one genetic study.

To investigate the statistical significance of the interactions among proteins encoded by the module genes, we analyzed these genes by using DAPPLE. The results showed 230 out of the 238 module genes participated in the direct network (S2 Fig), and the direct PPI network of module genes had significantly more edges than expected by chance (*P* = 9.9 × 10^−5^), which means the network formed by the module genes were statistically significantly connected.

To explore the biological function of the module genes, pathway enrichment analyses were conducted for 238 merged module genes, 68 MGS module genes, 29 Affy6 module genes and 146 Affy500K module genes respectively. The results were listed in Table 3. The pathways enriched by 238 merged module genes involved several signaling pathways (such as neurotrophin signaling pathway, VEGF signaling pathway), biological processes related with cellular adhesion, regulation of actin cytoskeleton, leukocyte transendothelial migration and regulation of protein metabolism, modification and ubiquitination, and cellular component of synaptosome. In addition, the analyses of the module genes from three separate datasets enriched an additional signaling pathway (GnRH signaling pathway) and one biological process (antigen processing and presentation).

**Table 3:** Enriched KEGG pathways and GO terms by module genes.

Gene Source^a^	Category	Term	N (X)^b^	FDR^c^
Merged	KEGG	hsa04670:Leukocyte transendothelial migration	118 (15)	8.32×10^−5^
Merged	KEGG	hsa04722:Neurotrophin signaling pathway	124 (14)	0.0011
Merged	KEGG	hsa04370:VEGF signaling pathway	75 (11)	0.0024
Merged	KEGG	hsa04810:Regulation of actin cytoskeleton	215 (17)	0.0058
Merged	KEGG	hsa04510:Focal adhesion	201 (16)	0.0114
Merged	KEGG	hsa04530:Tight junction	134 (13)	0.0154
Merged	KEGG	hsa04910:Insulin signaling pathway	135 (13)	0.0166
Merged	KEGG	hsa05200:Pathways in cancer	328 (22)	0.0019
Merged	KEGG	hsa05214:Glioma	63 (10)	0.0044
Merged	KEGG	hsa05222:Small cell lung cancer	84 (10)	0.0478
Merged	GO CC	GO:0019717~synaptosome	85 (10)	0.0065
Merged	GO BP	GO:0051247~positive regulation of protein metabolic process	243 (17)	0.0016
Merged	GO BP	GO:0032270~positive regulation of cellular protein metabolic process	233 (16)	0.0047
Merged	GO BP	GO:0031399~regulation of protein modification process	295 (17)	0.0196
Merged	GO BP	GO:0031400~negative regulation of protein modification process	119 (11)	0.0229
Merged	GO BP	GO:0031396~regulation of protein ubiquitination	100 (10)	0.0358
Merged	GO BP	GO:0031401~positive regulation of protein modification process	187 (13)	0.0497
Affy500K	KEGG	hsa04670:Leukocyte transendothelial migration	118 (11)	6.8×10^−4^
Affy500K	KEGG	hsa04722:Neurotrophin signaling pathway	124 (10)	0.0096
Affy500K	KEGG	hsa05200:Pathways in cancer	328 (15)	0.0133
Affy500K	KEGG	hsa04530:Tight junction	134 (10)	0.0180
Affy6	KEGG	hsa05214:Glioma	63 (6)	0.0026
Affy6	KEGG	hsa04912:GnRH signaling pathway	98 (6)	0.0234
MGS	GO BP	GO:0019882~antigen processing and presentation	83 (6)	0.0422

^a^ Merged denotes all genes of the merged modules from MGS, Affy6 and Affy500K.

^b^ N is total number of genes in the pathway or GO term. X is number of input genes which is mapped to the pathway. Only pathways or GO terms with N < 350 were shown.

^c^ FDR is the Benjamini & Hochberg-adjusted *P*-value. Only pathways or GO terms with FDR < 0.05 were shown.

CC: cellular component; BP: biological process.

**Fig 2:**
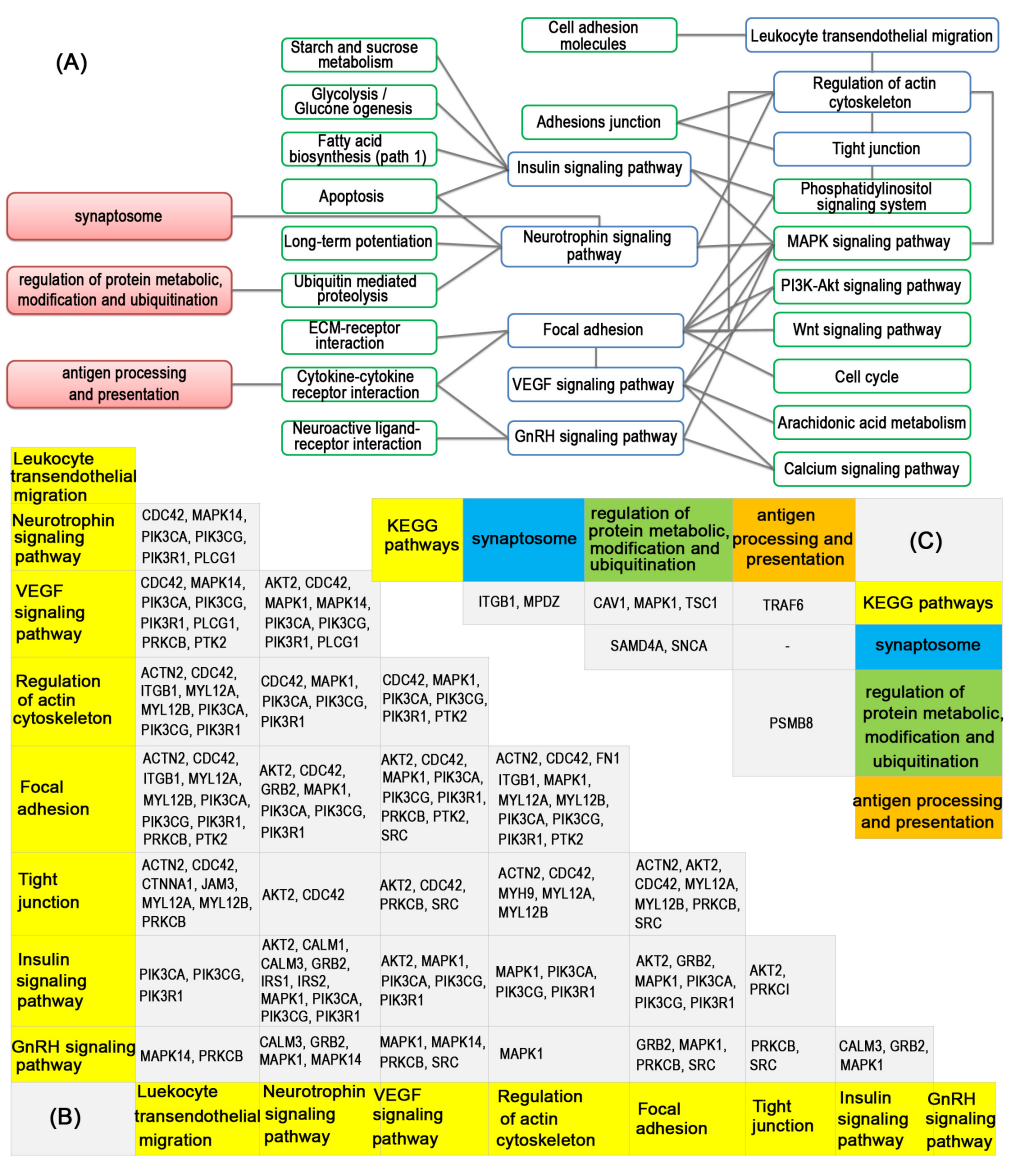
Crosstalk analysis of enriched pathways/GO terms. Panel A shows the connections among the enriched pathways/GO terms. Blue squares are enriched KEGG pathways, green squares are their connected pathways from KEGG, red squares are enriched GO terms. Panel B shows the shared genes between the first group of enriched pathways. Panel C shows the shared genes between four groups of enriched pathways/GO terms. The genes in groups with more than one pathway/GO term were combined for the shared gene analysis.

To further understand the functional connections between these enriched pathways/GO terms, a crosstalk analysis was performed for them. According to their function and shared genes, the enriched pathways/GO terms (except three cancer related pathways) were classified into four groups as shown in Fig 2. The first group included eight pathways from KEGG, which connect with many basic signaling pathways, such as MAPK signaling pathway, PI3K-Akt signaling pathway, and calcium signaling pathway. Furthermore, genes *MAPK1*, *CDC42*, *PIK3CA*, *PIK3CG* and *PIK3R1* were commonly shared by most of the eight pathways. The second group was synaptosome, which shares genes *ITGB1* and *MPDZ* with the first group. The third group included six GO biological processes related with regulation of protein metabolic, modification, and ubiquitination, which are functionally related with neurotrophin signaling pathway through pathway ubiquitin mediated proteolysis. All these six GO terms involved several proteasome related genes, including *PSMA2*, *PSMB6*, *PSMB7*, *PSMB8*, *PSMC1* and *PSMD14*, and ubiqutin C (*UBC*). The last group was antigen processing and presentation, which involves several immune related genes, such as *HLA-A*, *B2M*, *CD1D* and *TAP2*. The shared genes between these four groups of pathways/GO terms were shown in Fig 2 panel C.

## Discussion

Genetic data of schizophrenia has been accumulated rapidly over the past several years. In this study, we conducted a network-based analysis on three independent schizophrenia GWAS datasets to explore the joint effects of multiple genetic association signals on schizophrenia. After merging the modules identified separately from the three GWASs, 79 modules enriched with robust genetic signals in GWAS datasets were screened out, and 238 genes were involved in the identified modules. These modules and module genes could provide potential candidate genes, gene-gene interactions and molecular pathways involved in schizophrenia pathogenesis.

Of the 238 module genes, seven schizophrenia candidate genes had been collected in either GWAS Catalog or SZGene. Since the network-based analysis aims to search for module genes, which is a combination of several jointly GWAS signals, some genes with bigger *P*-value may also be selected. For example, among the seven replicated genes, although most of them had *P*-value < 0.05 in at least one of three SZ GWAS datasets, gene *VAT1L* had *P*-value > 0.05 in all three SZ GWAS datasets. The identification of *VAT1L* by our network-based analysis might be due to its connection with another two genes with *P*-value < 0.05 (*SEPT3* and *ARPC5L*). One of the seven genes, *MYH9*, had been analyzed as a candidate gene for schizophrenia in Japanese population [48], which was a three-stage case-control association study. In the first and second stages, the authors found a potential association of *MYH9* with schizophrenia (allelic *P*-value = 0.047). In the third stage, however, they could not replicate the result in a larger sample size. In another study conducted in Taiwanese sample, *MYH9* was found as a vulnerable gene for neuropsychological defined subgroups of schizophrenia patients (*P*-value = 0.0059 with haplotype analysis) [55]. So far, it is still arguable for *MYH9* about its susceptibility to schizophrenia, especially in Caucasian population. In addition, considering the possible shared genetic variants between SZ and BD, we also investigated how many identified module genes had been reported their susceptibility to BD. One of the results was *MAD1L1* gene, which also has been clearly reported its association with SZ by GWAS (*P*-value < 5 × 10^−8^) [44–46]. Another interesting gene is *ITIH1*, which had *P*-value = 1.03 × 10^−5^ in Affy500K, and was involved in an overrepresented interaction (*ITIH1*—*TIAM1*) in Affy500K modules. Notably, *ITIH1*, *ITIH3* and *ITIH4* belong to a family of serine protease and are arranged in the order of *ITIH1*-*ITIH3*-*ITIH4* on chromosome 3p21. Currently, ITIH molecules have been found to play a particularly important role in inflammation [56]. In addition, the region *ITIH3*-*ITIH4* has been identified to be susceptible to schizophrenia by GWAS (*P*-value = 7.8 × 10^−9^) [11]. In the study by He *et al*., they found three out of six SNPs they tested within the *ITIH1*-*ITIH3*-*ITIH4* genomic region were significantly associated with SZ in the Han Chinese population (the strongest SNP was rs2710322 with allelic *P*-Bonferroni = 0.0018) [57]. These lines of evidence implied that *IT1H1* might be not only associated with BD but also SZ. Among the unreported module genes, it is noteworthy that *DGKI* gene had gene *P*-value < 0.05 in all datasets (gene *P*-value in MGS = 3.4 × 10^−2^, in Affy6 = 2.7 × 10^−3^, in Affy500K = 2.17 × 10^−4^). In addition, the interactions between *DGKI* and *VHL* and *PSMA6* genes (*PSMA6—VHL—DGKI*) were overrepresented in Affy500K modules. Actually, *DGKI* has been reported to be related with schizophrenia at gene level test (gene-wide *P*_min_ = 6.7×10^−4^) [53].

Among the 238 module genes, there were 155 genes with *P*-value < 0.05 (65%) in any of MGS, Affy6 or Affy500K dataset. To check the *P*-values of our 238 module genes in a larger dataset, we calculated the gene *P*-values in SCZ2. Considering the huge number of SCZ2 SNPs, besides VEGAS, we also used GATES [27] to calculate the gene *P*-value in parallel. The results were shown in S1 Table. In summary, among all module genes, there were 88 and 61 genes with *P*-value < 0.05 by using VEGAS and GATES respectively, which contained totally 93 genes (39.1%), including several possible candidate genes mentioned above, such *ITIH1*, *DGKI*.

As one of the first group of pathways, neurotrophin signaling pathway has been reported to be associated with schizophrenia in several analyses [24, 25]. Neurotrophins are a family of trophic factors involved in differentiation and survival of neural cells [58]. The dysfunction of neurotrophin signaling pathway can affect axonal outgrowth, axonal guidance and synapse formation (from KEGG), which is functionally related with the second group of enriched GO term synaptosome. There is considerable evidence showing that abnormalities of synapse connectivity, synaptic transmission and synapse development contribute to the pathogenesis of schizophrenia [59, 60], so the enrichment of synaptosome is consistent with previous reports. Another interesting pathway is regulation of protein ubiquitination. The ubiquitin proteasome system has been identified as a canonical pathway associated with several neuropsychiatric disorders, including Alzheimer’s [61], Parkinson’s [62] and bipolar disorder [63]. Recently, Maria *et al*. have reported the abnormalities of ubiquitination system in schizophrenia by using gene expression analysis [64]. The evidence supported the association of this pathway with schizophrenia. The enrichment of antigen processing and presentation is also consistent with the finding of the association of genes in MHC region with schizophrenia in several GWASs of schizophrenia [11, 12]. Meanwhile, the shared gene analysis between different groups of pathways could reveal some important connected genes. *PSMB8* and *TRAF6*, both of which were shared by two groups of pathways and identified by two GWAS datasets separately, were two notable examples. *PSMB8* encodes proteasome, which cleaves peptides in an ATP/ubiquitin-dependent process in a non-lysosomal pathway [65]. An essential function of a modified proteasome, the immunoproteasome, is the processing of class I MHC peptides [66]. *TRAF6* encodes TNF receptor-associated factor 6, E3 ubiqutin protein ligase, which functions as a signal transducer in the NF-kappaB pathway that activates IkappaB kinase (IKK) in response to proinflammatory cytokines, and also interacts with ubiquitin conjugating enzymes catalyzing the formation of polyubiquitin chains [67]. Thus, these two genes are related with both immune system and protein ubiquitination process and may play important functions in these pathways. In summary, the four groups of enriched pathways/GO terms were potentially associated with schizophrenia, and crosstalks among them revealed they were connected with each other through some shared genes and basic signaling pathways, which may implement the neural and immune related functions and contribute to the pathogenesis of schizophrenia.

Compared with previous network-based analyses for schizophrenia [21, 24, 25], our analysis included more GWAS datasets and merged them into three groups according to genotyping platforms. Cross evaluation among three groups at the module level could improve the reliability of results. In addition, our analysis pipeline used more accurate gene *P*-value calculation and in-depth functional analysis for the module genes. We also compared our module network with two previous related studies. At the gene level, our module genes shared 12 genes with Jia et. al [21] and eight genes with Yu et. al [24] (the shared genes were shown in S1 Table). At the edge level (protein-protein interaction) our module network only share one edge with Jia et. al [21] (VHL (pp) FN1) and one edge with Yu et. al [24] (SRC (pp) GRB2). However, at the pathway level, our results validated several pathways enriched by other analyses, such as neurotrophin signaling pathway by [24] and [25], tight junction by [21] and [24], antigen processing and presentation by [25], which was also validated by another PPI network analysis paper about the top genes of schizophrenia [68]. Furthermore, one of the enriched GO terms of our analysis, “regulation of protein ubiquitination”, was firstly identified by network-based analysis, which was also validated by other types of studies [64]. These results would provide new insights and hypothesis for further study. Additional experimental replication and verification are required in future genetic, gene expression and molecular functional studies.

In conclusion, our study suggests that network-based analysis of schizophrenia GWASs is a useful method that could identify new susceptible genes, gene interactions and molecular pathways for schizophrenia. Our findings emphasize a central role for neural and immune-related pathways in the etiology of schizophrenia, and provide several candidate pathways and genes associated with schizophrenia, which would facilitate the understanding of genetic mechanism of schizophrenia.

## Supporting Information

S1 Fig Workflow of network-based analysis of GWAS data to identify functional modules for schizophrenia. (PDF)

S2 Fig The direct network formed by the module genes from DAPPLE. (PDF)

S1 Table Annotation for network module involved genes. (XLSX)

S2 Table PPI pairs involved in the identified modules for schizophrenia. Number of repeat denotes how many modules involved the interaction.
(XLSX)
