# Supplementary figures and images for: Network-Based Analysis of Schizophrenia Genome-Wide Association Data to Detect the Joint Functional Association Signals
Source: PLoS One. 2015 Jul 20;10(7):e0133404. doi: 10.1371/journal.pone.0133404 (PMC4508050; doi:10.1371/journal.pone.0133404)

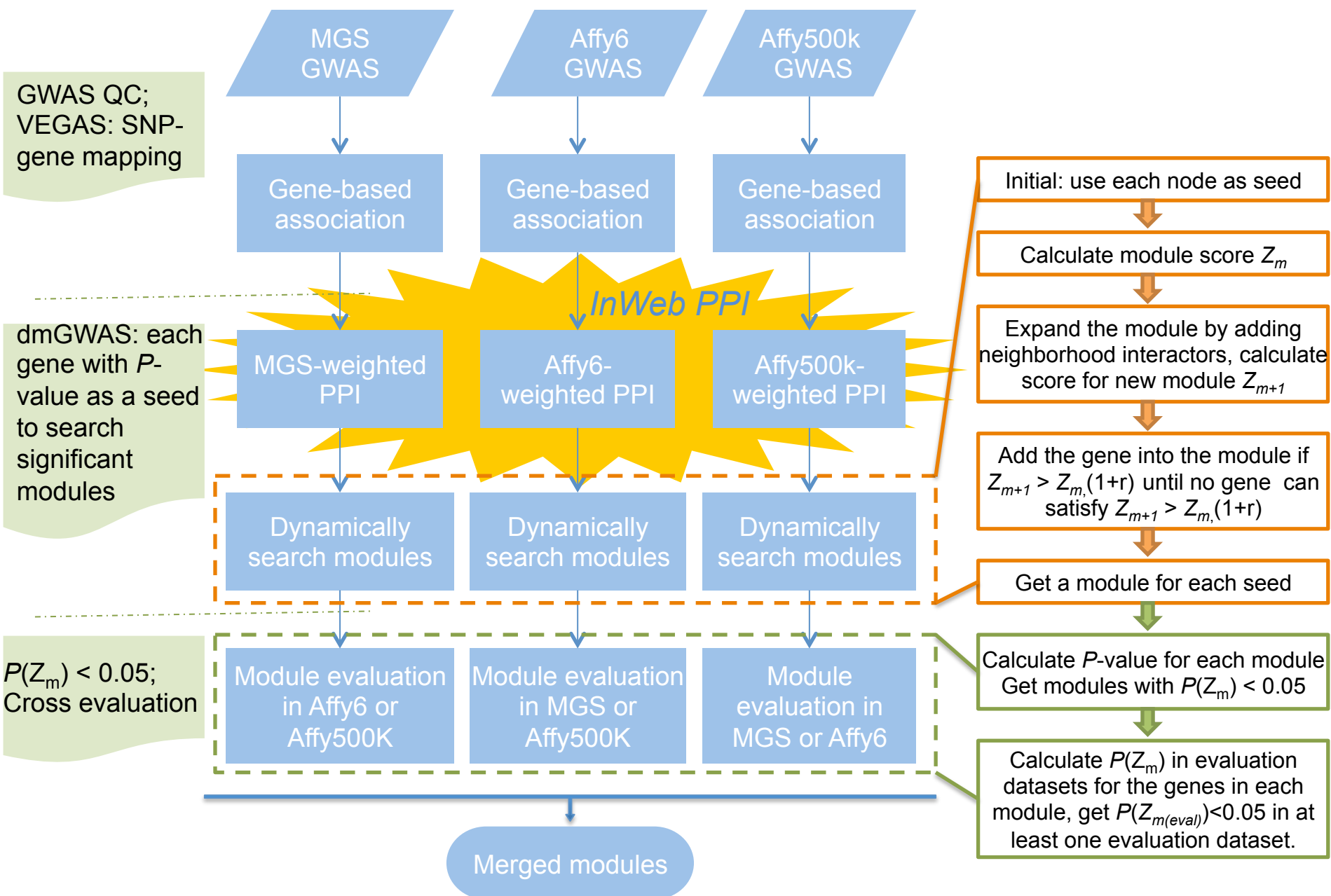

Supplement: S1 Fig — (PDF) [file pone.0133404.s001.pdf]

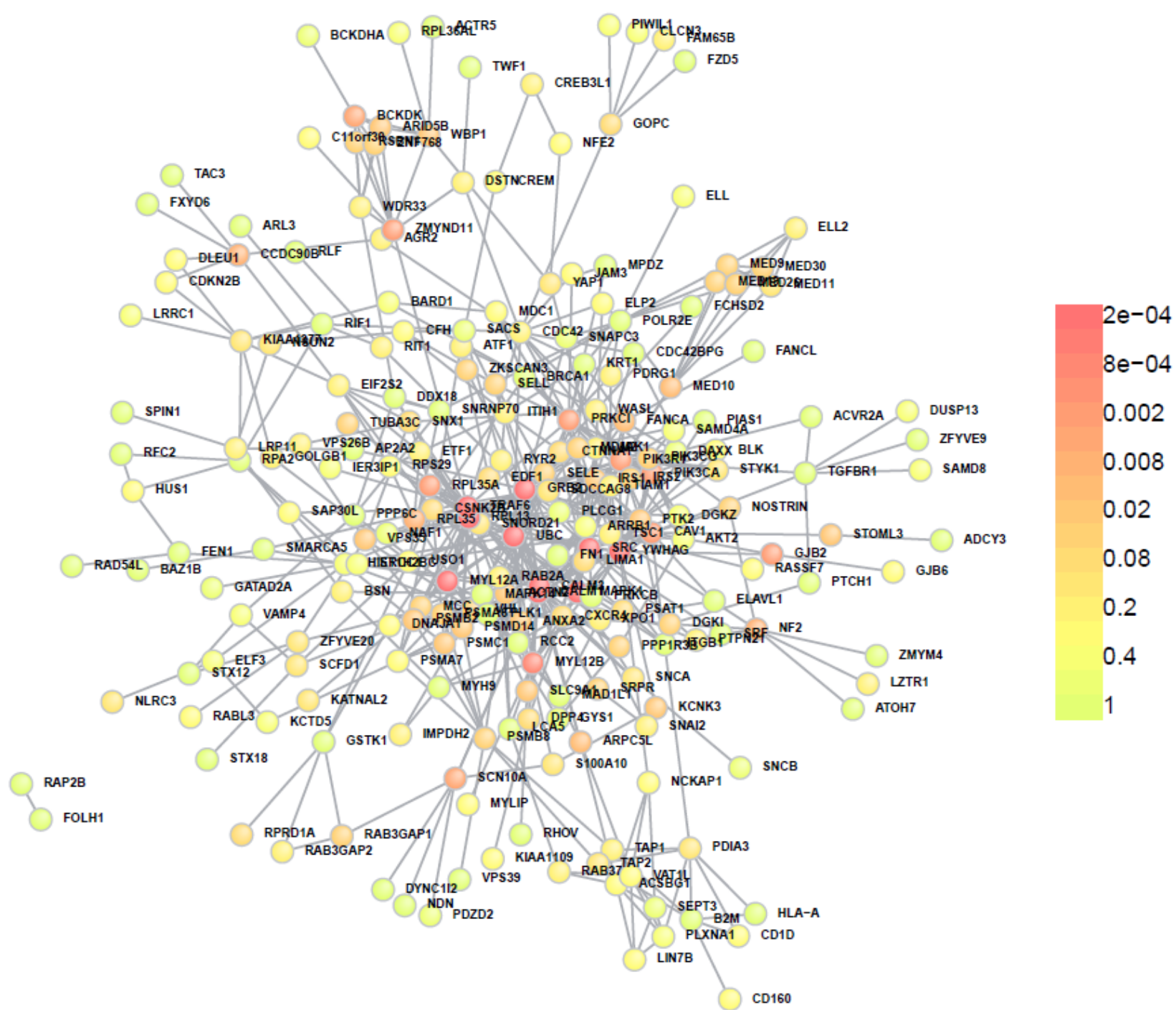

Supplement: S2 Fig — (PDF) [file pone.0133404.s002.pdf]
